# RGB Plots as a Tool for the Simultaneous Visualization of Multiple Data Layers in a Two Dimensional Space

**DOI:** 10.1371/journal.pone.0102903

**Published:** 2014-07-16

**Authors:** Yair Suari, Steve Brenner

**Affiliations:** Department of Geography and Environment, Bar-Ilan University, Ramat-Gan, Israel; University of Vigo, Spain

## Abstract

Visualization of multidimensional data helps in understanding complex systems and environments. We present here a red, green, blue (RGB) visualization method that can serve to display environmental properties. The saturation of each color is used to represent the concentration of a given property. The implementation of that figure is illustrated through visualization of three dissolved inorganic nutrient concentrations along a vertical transect of the Mediterranean, as well as through a vertical time series of three phytoplankton group cell numbers. The RGB figures show well known properties of the water column. In addition, they reveal some lesser-known properties, such as regions in shallow water in which the ratio of phosphorus and silica to nitrogen is high, and a deep eukariotic phytoplankton community. Visualization of such data is usually performed with three separate contour or surface plots, and occasionally two properties are presented as an overlay in a single figure. The RGB figure offers a better way to visualize the interactions among the three separate plots than is commonly available.

## Introduction

Classification and representation of multidimensional data are of great scientific interest. Methods such as the principal component analysis and k-means classify the data while losing the original data values. Cawthon et al. [1] explored the mapping of projected climate change and its uncertainty. A visualization method of four dimensional data in two dimensional space was developed, presenting data values by color (hue) and uncertainty by saturation. Roederer et al. [2] and Saadatinejad et al. [3] addressed the problem of displaying multidimensional data in two dimensional space by using the color matrices of the RGB model, thus showing raw flow cytometry results and seismic data through color saturation, respectively.

Currently all the visualization methods are limited to less than four simultaneous dimensions, including, up to two independent variables and one dependent variable. Following growth in the volume of data [4] as well as the increase in data complexity, new multidimensional data visualization methods must be developed to facilitate our understanding of complex systems.

The marine ecosystem is composed of diverse organisms that differ along various dimensions, such as taxonomy, function, or size. Moreover, the ecosystem is affected by many environmental factors such as temperature, salinity, light intensity, and nutrient concentration.

We illustrate the RGB representation methodology through the visualization of dissolved inorganic nutrient concentrations along a vertical transect of the Mediterranean. We focus on the macro nutrients: Nitrate (NO_3_), Phosphate (PO_4_)_,_ and Silica (SiOH_4_). Much scientific interest has been directed at the concentration ratios between these substances since Redfield [5] discovered that elemental C:N:P ratios in seawater are constant (106∶16∶1) and equal to the same elemental ratios in plankton. An inspection of the molar ratios of dissolved nutrients allows us to estimate the limiting nutrient [6]–[9]. The dissolved N:P ratios (22∶1) in the eastern Mediterranean diverge substantially from the Redfield ratio and therefore they raise specific interest [10], [11] that can be addressed through the use of RGB visualizations. Unlike nitrogen and phosphorus which are vital to all phytoplankton groups, silica is consumed by diatoms only.

We also apply the RGB visualization to a vertical time series of three phytoplankton group cell numbers. The phytoplankton community structure is greatly influenced by environmental conditions. Due to nutrient poor (oligotrophic) conditions in the eastern Mediterranean, cell sizes are smaller [12] and eukaryotic phytoplankton (Euk) relatively scarce [13]. The eukaryotic phytoplankton cells are usually larger than those of other phytoplankton groups and therefore their presence stimulates a more efficient food web [14] and might enhance the rate of carbon export to the deep ocean [14].

When oceanographic data are displayed graphically, geographic location and time of sampling often serve as independent variables, whereas quantitative variables, such as nutrient concentration or cell number, serves as the depended variable. The standard methods using a single dependent variable are insufficient for multidimensional representation and interpretation.

Here we present two figures of examples of multidimensional data-sets on computer screen or paper, using the qualities of the RGB color model. The dimensions consist of two in-dependent variables (axes of the plot) and three dependent variables represented by the color saturation. This method enables a more thorough representation of the marine environment than the methods currently used and facilitates the interpretation of the data by revealing the interdependence between the variables.

## Materials and Methods

### Ethics statement

The sampling site is located at an area which is not protected and no sampling permit is required. The field studies did not involve endangered or protected species.

The RGB representation of two in-dependent and three dependent variables, was applied to two different types of data: Mediterranean nutrient concentrations and phytoplankton group biomass. A short description of the data source is also provided. Data analysis was performed using the Matlab 8.1 commercial software package [15].

The first analysis was based on the gridded MEDATLAS [16] climatological nutrient concentrations. The horizontal resolution was 0.2° and the vertical resolution ranged between 10 m for shallow water and 500 m for water deeper than 1500 m. These data were produced by an optimal interpolation technique and constructed from historic Mediterranean nutrient samples, covering the years 1864–2002.

**Figure 1:**
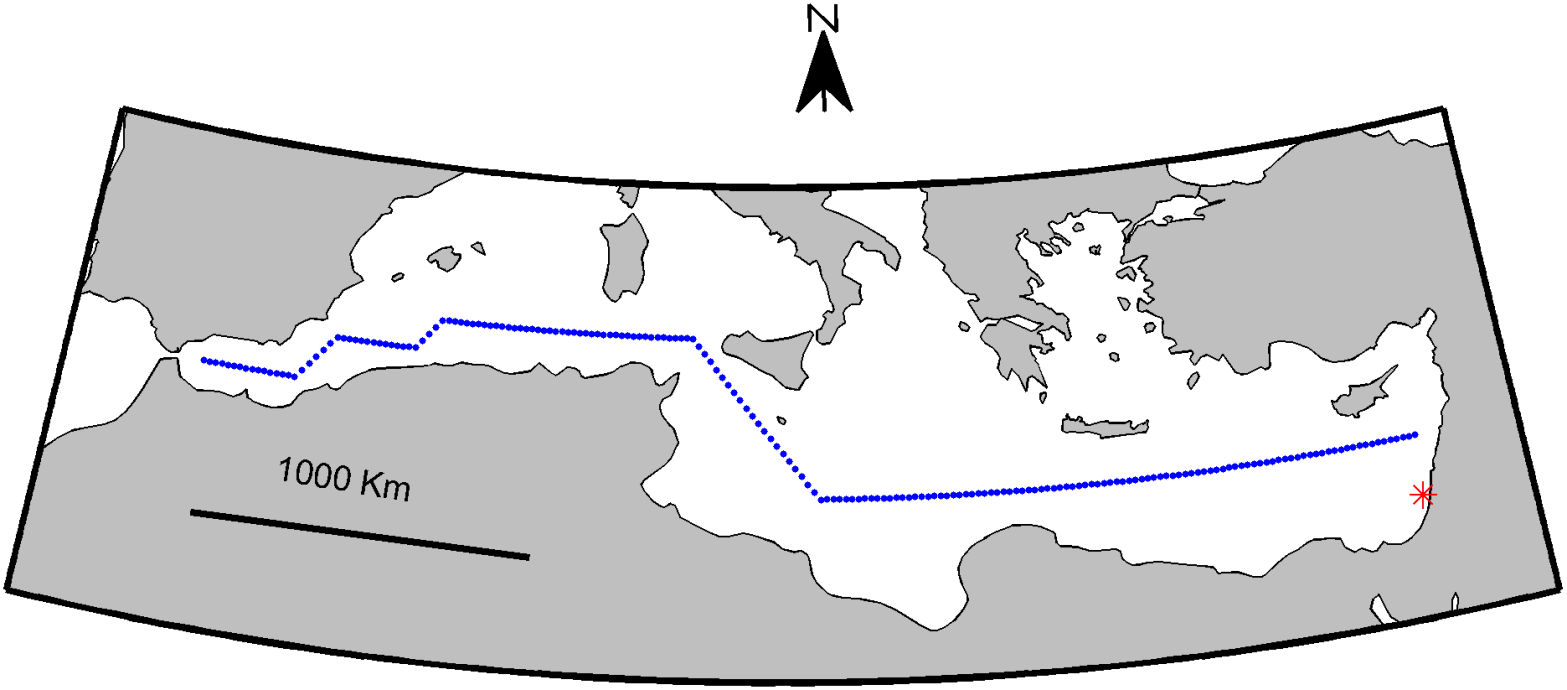
Map of presented data points. Nutrient concentration profiles were extracted from the MEDATLAS database [16] along the blue line the plotted on the map to create the RGB nutrient Figure (Figure 2). The red asterisk at the eastern coast represents the sampling point for the phytoplankton taxonomic composition, as presented in Figure 4.

Construction of an RGB figure from the MEDATLAS nutrient concentration fields (Equations 1–3) was done by extracting a vertical section along the Mediterranean (Figure 1) for each of the three nutrients (NO_3_, PO_4,_ and SiOH_4_). Concentration of each nutrient was normalized to the concentration range, assuming a minimal concentration of zero and dividing each grid point concentration by the maximal value found for that nutrient. For each depth (z) and distance (x) we calculated the square root of the normalized concentration. This calculation produced a saturation value for NO_3_ (red, R), PO_4_ (green, G), and SiOH_4_ (blue, B). Using the square root facilitated a more detailed view of the photic zone, in which the lowest nutrient concentrations are found and most of the primary production is performed. The color saturation values are given by
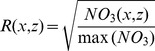
(1)

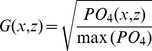
(2)

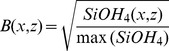
(3)


The second analysis used phytoplankton samples collected at the edge of the Israeli continental shelf (34.564°E, 32.217°N, Figure 1) during the first half of 2013. These samples were subjected to a taxonomic analysis conducted with Attune Acoustic Focusing Flow Cytometer as in Marie and Brussaard [17]. This method is commonly used in the analysis of marine phytoplankton, producing cell numbers for picoeukariots (Euk), *Synechococcus* (Syn), and *Prochlorococcus* (Pro) in seawater volume.

The RGB figure of the phytoplankton taxonomic composition was generated by normalizing the sample’s cell concentration in each specific taxon according to the range of concentrations for those taxa in all samples, thus representing the concentrations on a scale of zero to one. The normalized concentrations were then linearly interpolated to a Cartesian grid, in which time (t) and depth (z) of sampling were used as the X and Y axes, respectively, and the dependent variable was the normalized cell concentration. The interpolated fields were then used to construct the three RGB color components, as shown in equations 4–6.
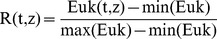
(4)

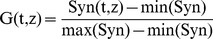
(5)

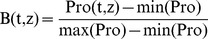
(6)


**Figure 2:**
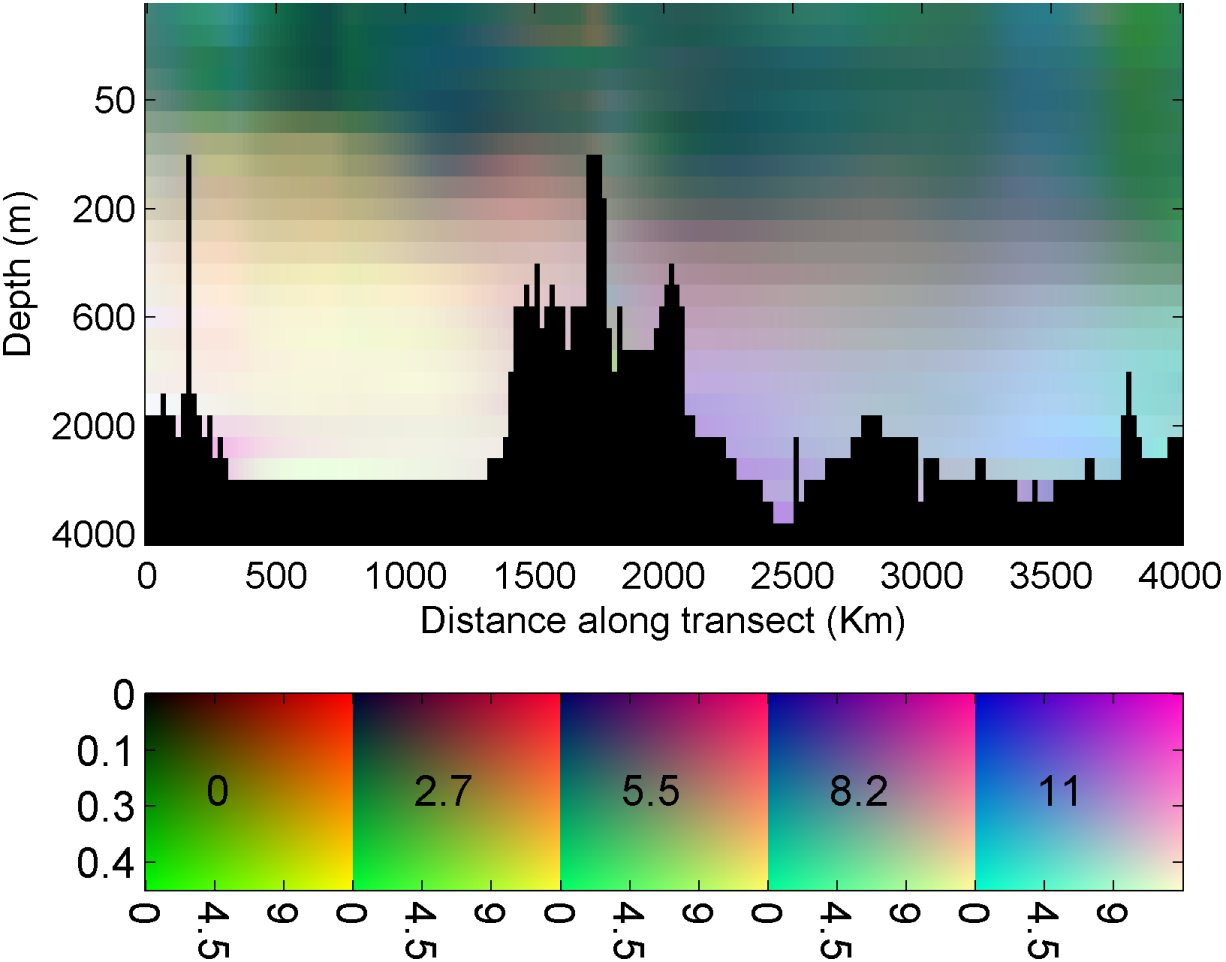
Top: RGB figure representing NO_3_ (red), PO_4_ (green) and, SiOH_4_ (blue) concentrations within an eastern Mediterranean transect. An RGB color scale is presented on the bottom right side with, the red saturation scale (NO_3_ concentration) on the Y axis, the green saturation (PO_4_ concentration) on the X axis, and a different blue saturation (SiOH_4_ concentration) in each rectangle (its value written within rectangles).

Many types of RGB scales exists, including the RGB triangle [18] that displayes RGB color combinations. Most RGB scales display only some of the color combinations, as all possible color combinations can only be displayed in a three-dimensional space. The RGB legends scale in the current manuscript was constructed by displaying continuous red and green saturation values as well as five saturation values for the blue color (Figure 2, bottom). Environmental concentrations were assigned to the scale axes.

## Results

The nutrient concentration RGB plot is shown in the upper panel of Figure 2. Well known features of the region are clearly illustrated, including the reduced surface nutrient concentration revealed by the darker shallow water, the West-to-East nutrient reduction [19] revealed by the darker eastern Mediterranean, and the increased N:P ratio in the deep water of the eastern Mediterranean [10], revealed by the redder deep water in this region. Other features shown on the RGB figure are the elevated proportions of phosphorus (green) in the eastern Mediterranean surface water, specifically at 0–200 m depth between kilometers 2800–3200 and 3700–4000 along the transect, and a patch of elevated silica (blue) proportion situated between them around kilometer 3500.

**Figure 3:**
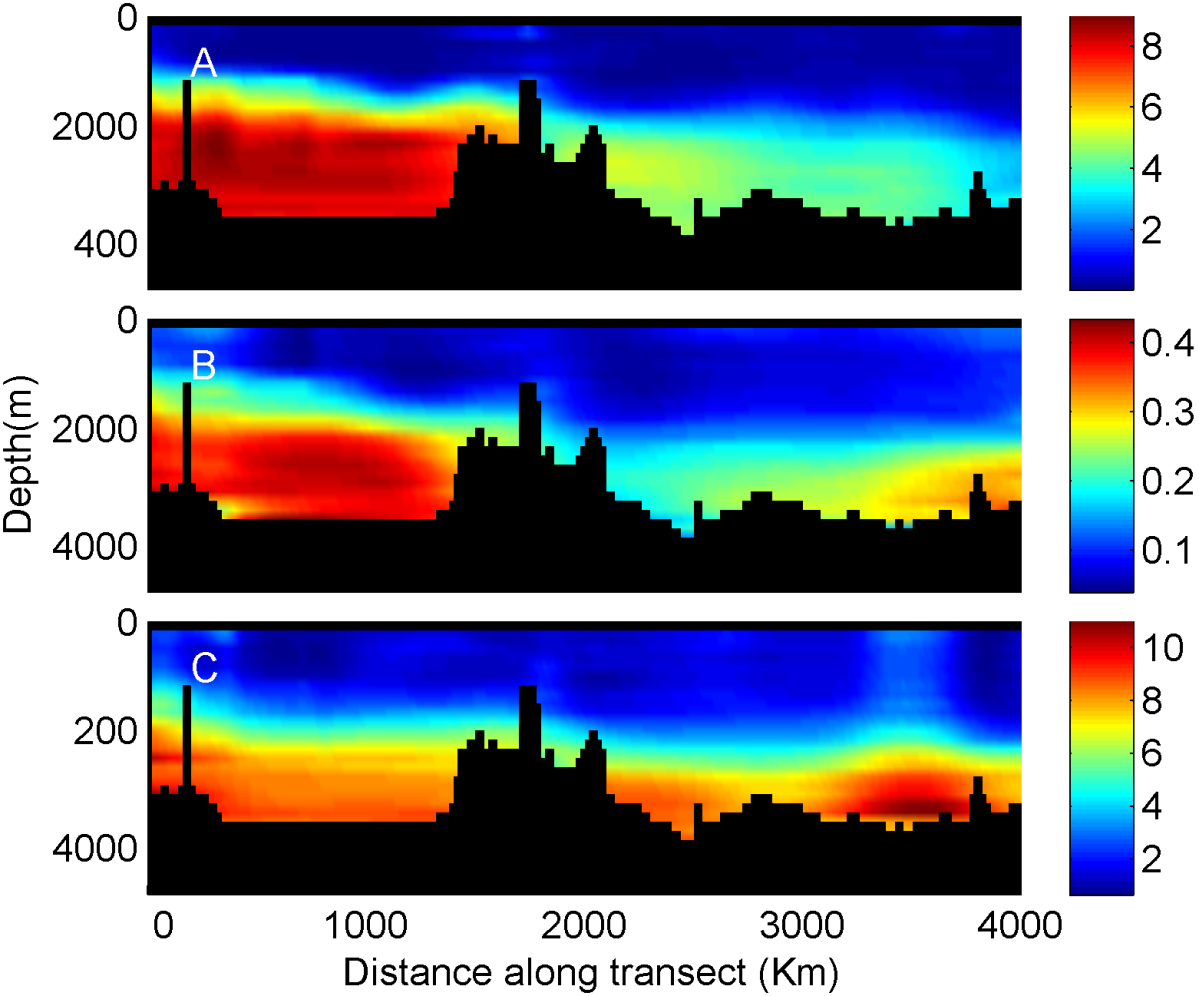
NO_3_ (A), PO_4_ (B), SiOH_4_ (C) vertical transects along the Mediterranean. Data source and transect location are described in Figure 1.

A more traditional visualization of the nutrient concentration data is displayed in Figure 3 where each nutrient is plotted separately (nitrate, upper panel; phosphate, middle; silicate, lower). Here the concentration gradients for each individual nutrient are better visualized but the concentration ratios cannot be easily resolved or interpreted.

**Figure 4:**
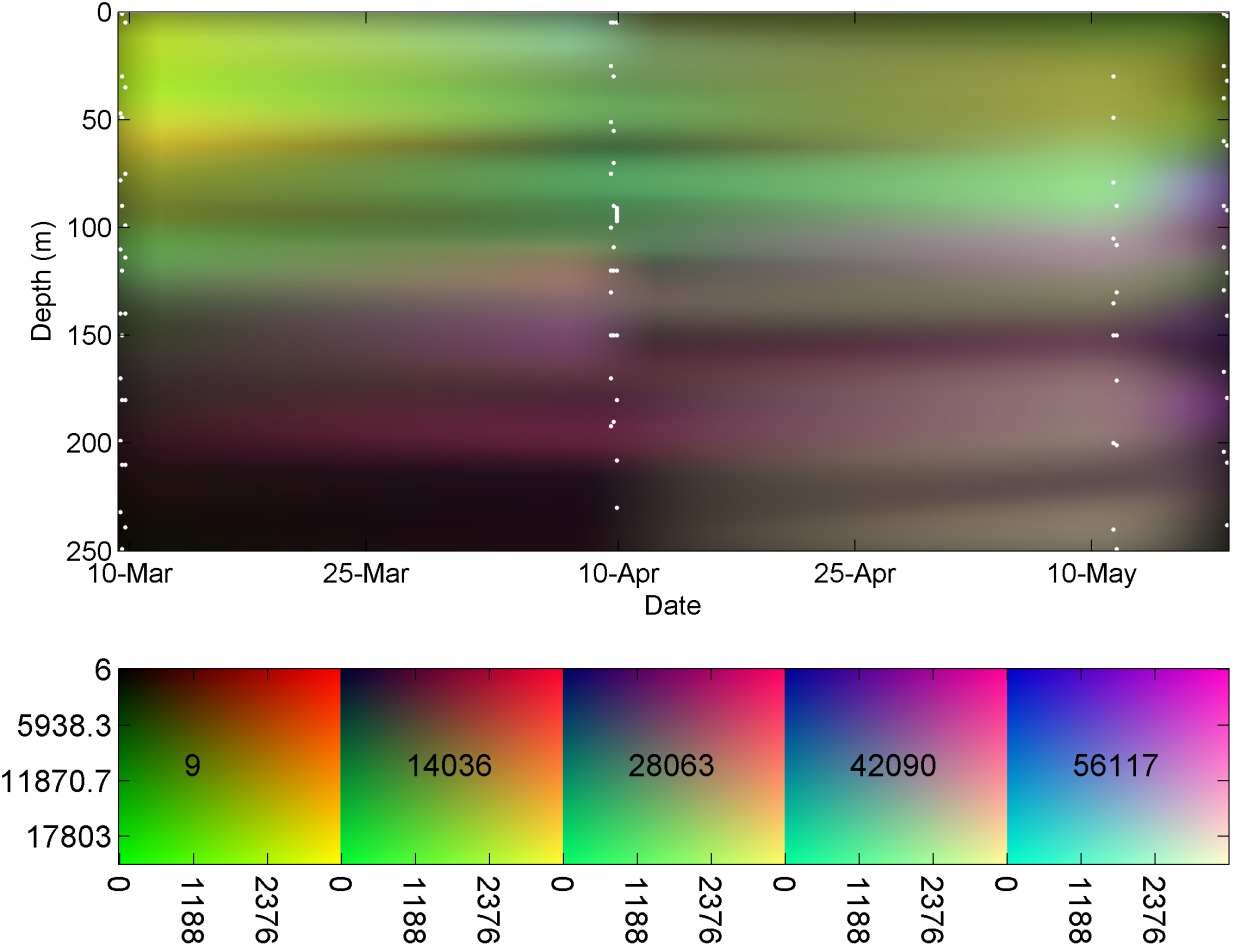
Vertical RGB time series of phytoplankton taxonomic composition Red, Green, and Blue stands for Eukariotic algae, Synechococcus, and Prochlorococcus respectively as presentedby the color-bar. Sampling time and depth are indicated by the white dots.

Figure 4 visualizes the phytoplankton community composition. The deep phytoplankton maximum is seen at ca. 50 m during late winter, deepening to ca. 100 m at late spring in the form of the brighter colors. An increase in the proportion of Pro in summer is also seen, in the form of bluer color on the right side of Figure 4 at a depth of ca. 80 m. Another feature revealed by the RGB representation is the existence of a Euk dominated phytoplankton community at depth of ca. 200 m throughout the period, which appears as a redder stripe at this depth.

## Discussion

The RGB method of analysis and presentation can help researchers identify patterns in multi-dimensional data and should be compared to conventional visualization of the same data in this context. Nutrient concentrations are commonly presented through a one dimensional vertical profile with the water depth on the Y axis and concentration on the X axis. This presentation is well suited for displaying the vertical gradient and the nutrient concentration range.

A more detailed presentation of nutrient concentrations is provided when interpolating the data to vertical transects or surface maps (Figure 3). Transects display detailed information about each nutrient’s concentration individually. To assess nutrient ratios at specific locations using such figures, one would have to switch one’s glance between the plots in Figure 3, a task that is most likely too difficult to be accurate.

Elemental nutrient concentration ratios are considered critical in determining the nutrient that most limits primary production [5], [7], [8], [20] and phytoplankton taxonomic composition [21]. Graphical visualization of the concentration ratio can be performed by vertical profiles or vertical transect contour plots of N:P ratios. Such representation reveals more details about the ratios but is still limited to comparison of two factors, preventing simultaneous evaluation of three or more variable ratios. Furthermore ratio plots only display the ratio, whereas the RGB figure visualizes both ratio and the concentration together. Note that Moore et. al. [9] displayed surface concentration maps of the major nutrients in which the NO_3_ concentration was divided by the Redfield ratio of 16. This presentation simplified the comparison of the NO_3,_ and PO_4_ surface maps but still forced the reader to switch between figures.

The presentation of phytoplankton community composition in Figure 4 can be compared to the traditional representation of similar data, such as Figures 9 and 11 in Siokou-Frangou et al. [22] (referred to as S-F). Comparing the current figure to this previous representation demonstrates the added value of the RGB method. In S-F Figure 9 a taxonomic time series similar to the one used to produce the current Figure 4 is presented in the form of a line plot for each taxon, with time at the X axis and concentration at the Y axis. The vertical structure of the water column must be omitted to enable such representation. The S-F Figure 11 presents a West-to-East transect along the Mediterranean, describing three taxonomic groups in the form of stacked vertical profiles. Depth is marked on the Y axis, concentration on the X axis, and a separate plot is used for each sampling station. To read and interpret this visualization one must switch glances between figures and compare taxonomic structure at different locations while disregarding the horizontal distance between samples. In both cases the reduction in the data presented is performed with the intention of enabling representation of three community composition components. Such data loss would be avoided using the RGB method.

## Summary and Conclusions

We presented a novel methodology for visualizing data containing multi-dimensional dependent variables in a single plot, facilitating interpretation of the data. The method is based on an RGB color model for simultaneous representation of three dependent variables. We demonstrated the usefulness of this methodology by displaying a vertical transect of inorganic nutrient concentrations along the Mediterranean as well as a vertical time series of marine phytoplankton cell numbers. The strength of the methodology is seen in its ability to display of trends of nutrient concentrations ratios and, of a deep Euk phytoplankton community. The authors could not find any scientific publication illustrating those phenomena.
